# Investigation on Anti-Autofluorescence, Osteogenesis and Long-Term Tracking of HA-Based Upconversion Material

**DOI:** 10.1038/s41598-018-29539-8

**Published:** 2018-07-26

**Authors:** Xiyu Li, Qin Zou, Wei Li, Haifeng Chen

**Affiliations:** 1 State Key Laboratory of Oral Diseases & National Clinical Research Center for Oral Diseases, West China Hospital of Stomatology, Sichuan University, Chengdu, 610041 China; 2 Analytical and Testing Center, Sichuan University, Chengdu, 610064 China; 3 Department of Biomedical Engineering, College of Engineering, Peking University, Beijing, 100871 China

## Abstract

Hydroxyapatite (HA) material will be long-standing once implanted in bone tissue of the body. It should be considered to endow the osteogenic HA material with traceable fluorescence to realize a lifelong *in vivo* tracking. We prepared and utilized lanthanides-doped HA upconversion material, and revealed for the first time that the lanthanides (ytterbium (Yb) and holmium (Ho)) co-doped HA upconversion material was suitable for long-term or lifelong *in vivo* tracking, the lanthanide ions doped in the HA matrix would not affect the biocompatibility and osteogenesis, and the tissue autofluorescence could be effectively avoided by the HA:Yb/Ho upconversion material. Also the distribution in bone and osteointegration with bone of the HA:Yb/Ho material could be clearly discriminated by its bright fluorescence under NIR irradiation. The upconversion characteristic of the HA:Yb/Ho material provides a feasibility and promising prospect for lifelong *in vivo* tracking, and has an advantage in revealing the material-tissue interrelation. The material has important clinical application value in addition to its usefulness for scientific investigation.

## Introduction

The mineral of human hard tissues is mainly composed of hydroxyapatite and closely related to the regeneration of bones and teeth^1–3^. Synthetic hydroxyapatites (HA) have been used to repair the bone defects or as fillers of the surrounding cavity of dental implants, and as bioactive component of tissue engineering scaffolds^4–6^. Successful clinical applications rely on the inherent affinity of HA to human tissues and its similarity to the apatite in bone and tooth. Especially the bioactivity of HA endows its bonding with new bone tissues. However, the HA material will be long-standing once implanted in human body accompanying the bone reconstruction and metabolism process^2^. So far, it remains unclear that how long the synthetic HA material will exist in bone tissue after implantation, and what’s its state during a lifelong period. Therefore, how to long-term trace the implanted HA material is still a technical and clinical challenge.

In recent years, various inorganic fluorescent materials have been developed including quantum dots^7,8^, gold nanospheres^9^, and rare-earth doped materials^10–12^. These materials have an advantage in overcoming the photobleaching and photoinstability of organic fluoresceins^13^ and in applications of detection^14^, cell imaging^15^, and targeted therapy^16^. The rare-earth doped upconversion materials can emit visible light when excited by near-infrared (NIR) light. They have drawn great attention because of their photostability, high contrast, and low toxicity^17,18^. However, there is few report respects to an investigation of these inorganic fluorescent materials on bone repair and reconstruction process. To trace a material involved in bone reconstruction process and in a lifelong period, the fluorescent material must own bone-bonding bioactivity and can overcome the interference of tissue autofluorescence in addition to good biocompatibility. Incorporating lanthanide ions into HA structure to endow HA with upconversion fluorescence will help to achieve the purpose of long-term tracing the implanted HA materials and revealing material-tissue interrelation. With the development of laser confocal microscopy, a combination of the lanthanides doped HA material and the confocal microscope is expected to provide new biomedical method and applications, and the NIR laser may also give deeper penetration *in vivo* than the UV light.

At present, the lanthanides (e.g. Yb/Ho) co-doped apatite including fluorapatite (FA) and hydroxyapatite (HA) should be the optimal candidate to meet such requirements. Compared to HA, the lanthanides doped FA reported by us and others normally shows stronger fluorescence intensity due to the low phonon energy of F^−^ ions and the more compact crystal structure^19,20^. And the F^−^ ions have also higher electronegativity (3.98) and smaller ionic radius (0.136 nm) than the -OH groups (3.51 and 0.176 nm) of HA^21^. However, the mineral of human hard tissues is mainly composed of HA rather than FA, and the F^−^ ions or the calcium binding with F^−^ ions may have potential risk leading to fluorosis or bone brittleness^22^. Hence, the lanthanides doped HA should be preferentially selected despite of its relatively lower fluorescence intensity than the lanthanides doped FA.

As we know, the HA matrix with a general formula Ca_10_(PO_4_)_6_(OH)_2_ has a stable hexagonal crystal structure belonging to P63/m space group, and the Ca^2+^ sites can be substituted by many other cations for various purposes^23,24^. The lanthanide ions have analogical radius as Ca^2+^ ions and high affinity to PO_4_^3−^ ions^25^, which can ensure the success of rare-earth doping.

**Figure 1:**
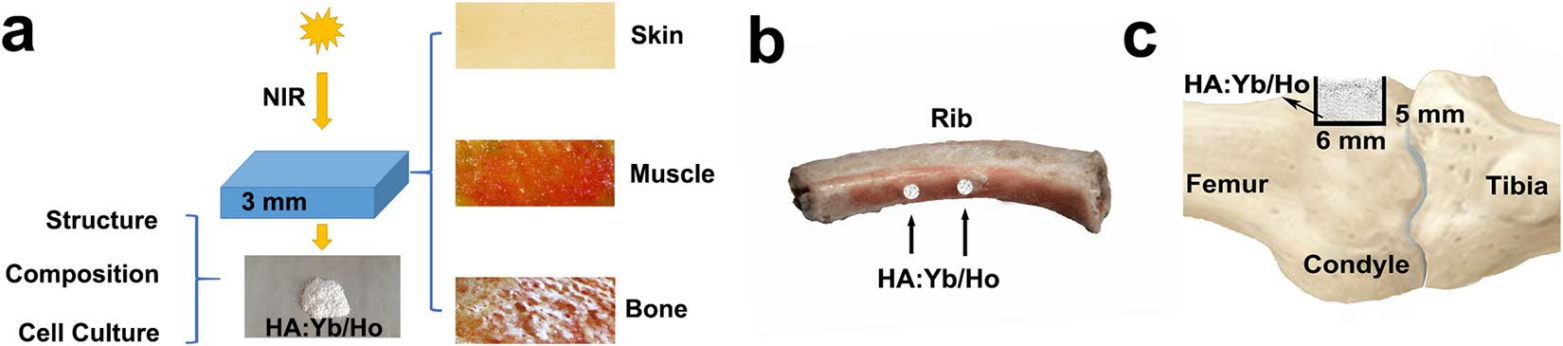
Diagram of HA:10Yb/1Ho powder covered by different pig tissues (**a**), filling in the drilled holes of pig rib (**b**), and implanted in defect of rabbit femoral condyle (**c**).

Substitution of lanthanide (Ln^3+^) ions for Ca^2+^ ions will cause Ca^2+^ vacancies in the apatite crystal structure due to the demand in charge balance^26^, which may affect the lattice parameters and structure. Besides, the lanthanides doping dosage may also influence the fluorescence property. In the study, lanthanides co-doped HA:Yb/Ho materials with varying dopant concentration were prepared by hydrothermal method. In addition to the analyses of their crystal structure, fluorescence characteristic and cytocompatibility, a selected HA:Yb/Ho powder was also used to detect the anti-autofluorescence property, osteogenesis and long-term tracking capacity, via being covered by different pig tissues, filling in the drilled holes of pig rib, and implanted in the defects of rabbit femoral condyles, as shown in Fig. 1. By the research, we attempt to reveal whether the lanthanides doped upconversion HA material is suitable for long-term or lifelong *in vivo* tracking, whether the lanthanide ions in HA matrix will affect the biocompatibility and osteogenesis, and whether the tissue autofluorescence will interfere the fluorescence tracking of the HA:Yb/Ho material.

## Results

**Figure 2:**
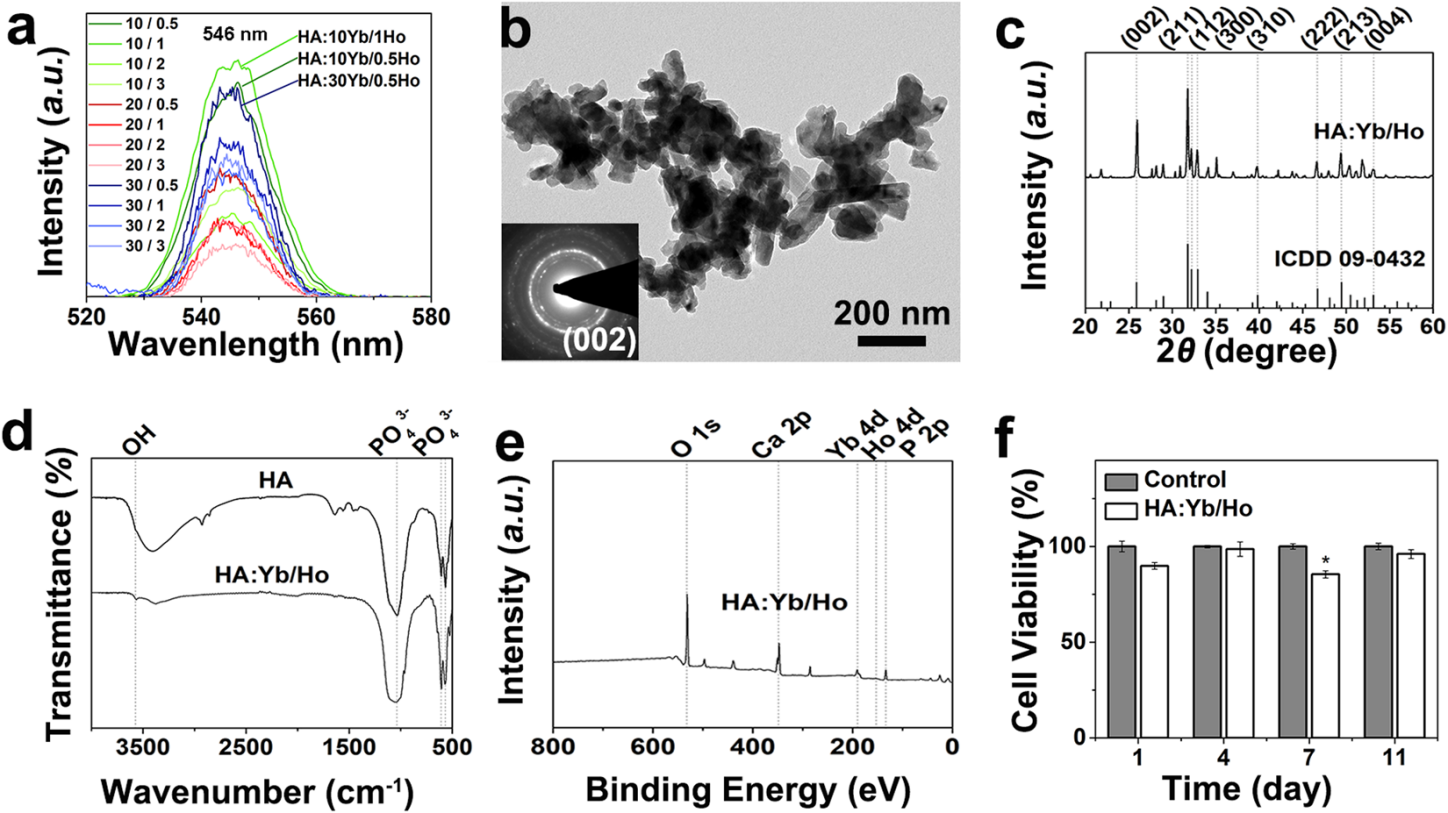
The emission spectra with various Yb/Ho doping concentrations of HA:Yb/Ho particles (**a**), and TEM image with SAED inset (**b**), XRD pattern (**c**), FTIR spectrum (**d**), XPS spectrum (**e**), and cell viability of HA:10Yb/1Ho particles with culture time (**f**), *p < 0.05.

Figure 2a shows the green emission spectra centered at 546 nm of the HA:Yb/Ho particles with various Yb/Ho doping concentrations under 980 nm NIR excitation. The emission intensities of HA:Yb/Ho with ratios of 10/1, 10/0.5 and 30/0.5 are the top three, much higher than the rest, and the HA:10Yb/1Ho owns the highest intensity. The bright green emission of HA:Yb/Ho is very stable and has good repeatability, indicating a tight coordination of the doping Ln^3+^ ions with the HA crystal structure. The morphology, crystal structure and composition of the lanthanides doped HA:10Yb/1Ho particles were examined by transmission electron microscope (TEM), X-ray diffraction (XRD), Fourier transform infrared (FTIR) spectroscopy, and X-ray photoelectron spectroscopy (XPS). After thermal activation at 700 °C for evoking the upconversion property, the HA:Yb/Ho particles show an irregular and agglomerated morphology (Fig. 2b) with a selected area electron diffraction (SAED) pattern of HA structure (inset). The XRD pattern in Fig. 2c shows characteristic peaks around 25.9°, 32°, 32.3°, 40°, 47°, 50°, and 53° corresponding to the (002), (211), (112), (310), (222), (213) and (004) lattice planes respectively, which match well with the hexagonal phase of classical HA (Ca_10_(PO_4_)_6_OH_2_, ICDD 09-0432). The measured lattice parameters of the HA:Yb/Ho are in good agreement with the data of pure HA, as shown in Table 1. Figure 2d shows the FTIR spectra of HA:Yb/Ho, which are similar to that of pure HA. The absorption peak at approximately 3570 cm^−1^ attributes to the typical stretching vibration of -OH groups, and peaks at 1043–960 cm^−1^ (asymmetrical stretching vibration) and 606–575 cm^−1^ (bending vibration) correspond to PO_4_^3−^ groups. The XPS spectrum in Fig. 2e demonstrates the presence of doping lanthanides in HA crystal structure. The binding energy data of Ca2p, P2p and O1s from XPS analysis for the HA:Yb/Ho are also listed in Table 1, showing a value slightly lower than that of pure HA. These results indicate that the entrance of Yb^3+^ and Ho^3+^ ions will slightly affect the ions interactions in HA lattice, but will not change the HA hexagonal crystal structure which is crucial for stable fluorescence emission of the doping ions. The ICP-OES data of HA:Yb/Ho show that the molar concentrations of Yb and Ho are 10.18% and 1.13% respectively, similar to the calculated composition of 10% Yb and 1% Ho.

**Table 1:** The lattice parameter and binding energy of HA and HA:Yb/Ho.

	a = b (nm)	c (nm)	Ca 2p (eV)	P 2p (eV)	O 1s (eV)
HA	0.942	0.688	347.0	133.0	531.0
HA:Yb/Ho	0.942	0.688	346.8	132.9	530.9

The cell proliferation/viability of MG63 cells cultured with 100 μg/mL dosages of the HA:10Yb/1Ho aqueous solution for 1, 4, 7, and 11 days was evaluated by a CCK-8 assay. The measured OD values at 1, 4, 7, and 11 days are 0.144, 0.398, 0.659 and 0.765 for the control and 0.130, 0.392, 0.563 and 0.735 for the HA:Yb/Ho material, respectively, illustrating a normal increased proliferation trend like the control. Figure 2f shows the viability of MG63 cells responding to the HA:Yb/Ho material, setting the control as 100% at different time point. The cell viability for the material group is generally comparable to the control at 1, 4 and 11 days (p > 0.05) except a slight lower than the control on day 7 (*p < 0.05). The CCK-8 assay indicates that the Yb/Ho-doped HA material has no obvious negative effect on the cell proliferation and viability, and can be used for further *in vivo* investigation.

An investigation on the interference of tissue autofluorescence to the upconversion luminescence of HA:Yb/Ho material was carried out and compared to that of downconversion HA:Tb material. For the HA:Tb powder which were covered by various pig tissues of approximately 3 mm in thickness, the tissues of fresh pig skin, muscle and bone display a blue, green or red autofluorescence under irradiation of UV, blue or green lights, respectively (Fig. S1 in Supplementary Information). However, there is no tissue autofluorescence under irradiation of 980 nm NIR light. The green fluorescence of the downconversion HA:Tb powder covered by these tissues cannot be observed on these tissues.

**Figure 3:**
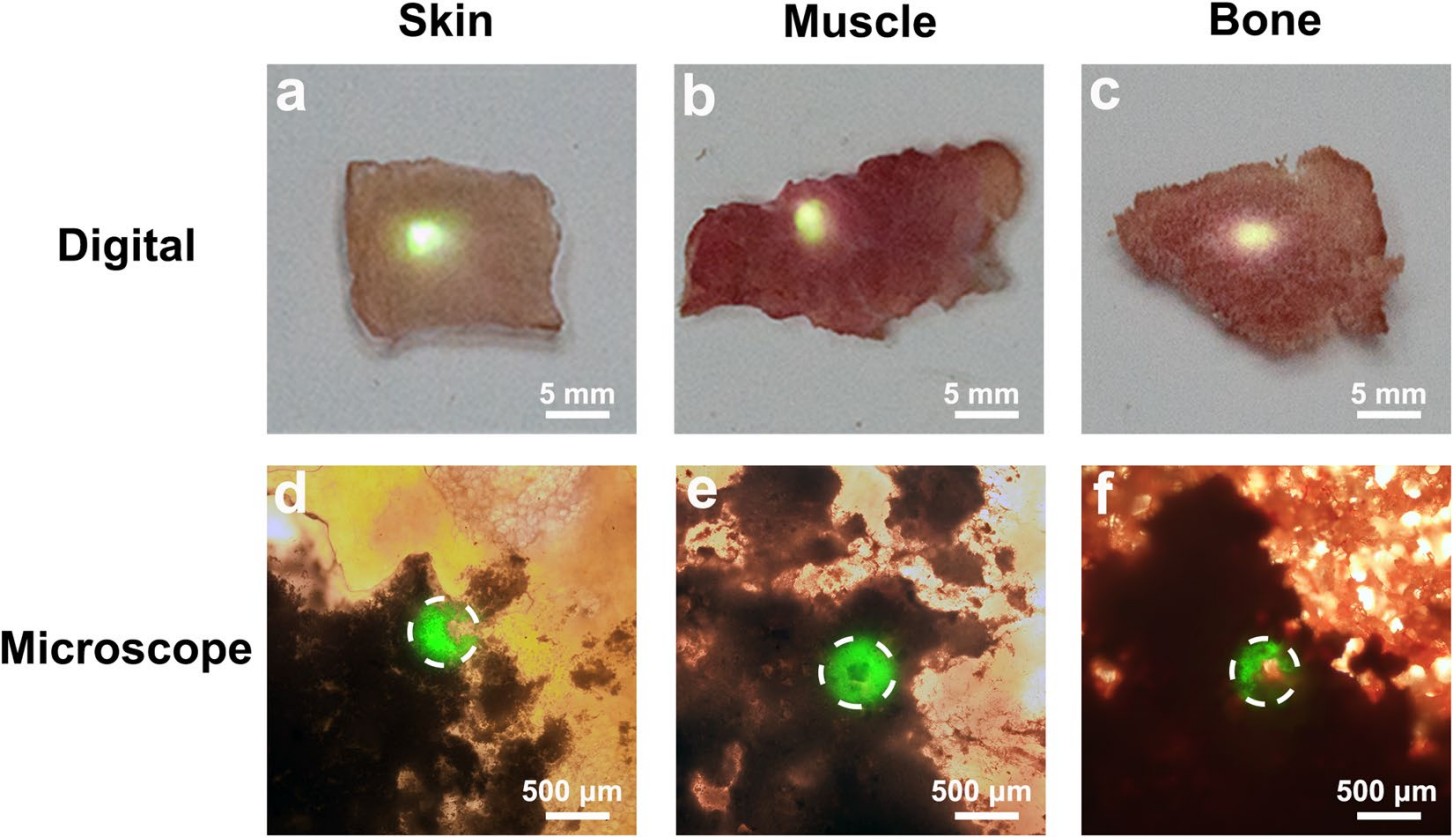
The digital (**a**–**c**) and microscopic (**d**–**f**) images of the upconversion HA:Yb/Ho powder covered by natural tissues of approximately 3 mm in thickness. The dotted white circles represent the NIR irradiation zone, and the bright and undisturbed green fluorescence is from the underlying upconversion HA:Yb/Ho powder under irradiation of 980 nm NIR light.

Figure 3 exhibits the digital and microscopic images of the upconversion HA:Yb/Ho powder covered by natural tissues of approximately 3 mm in thickness. The green fluorescence of the upconversion HA:Yb/Ho powder can be clearly observed on the tissues by naked eyes (a–c) and under inverted fluorescence microscope (d–f), which demonstrates that the NIR light has a deeper penetration in tissues, and the bright and undisturbed upconversion fluorescence of the HA:Yb/Ho material can effectively pass through the upper tissues under irradiation of the 980 nm NIR light. It indicates that the HA:Yb/Ho material has an advantage for *in vivo* tracking, because the tissue autofluorescence is not present under irradiation of 980 nm NIR light.

**Figure 4:**
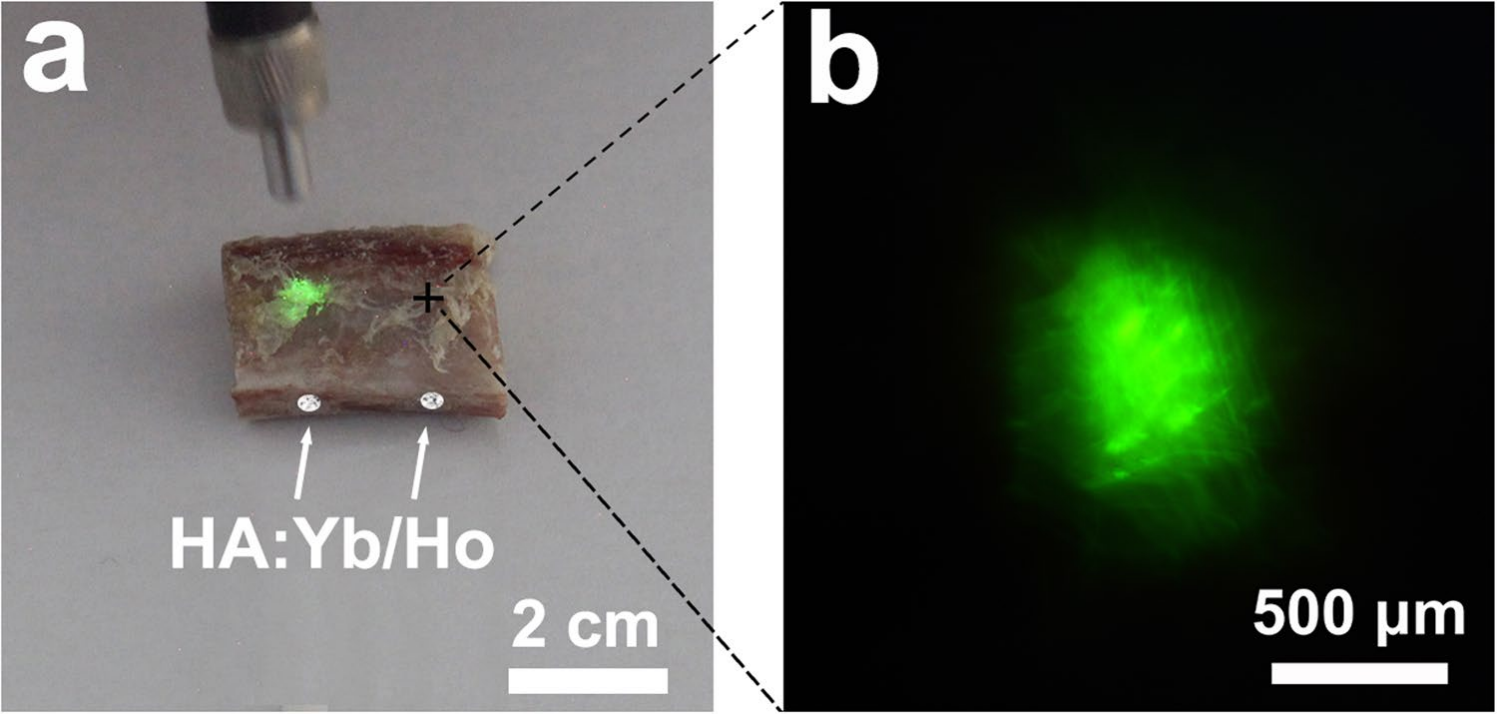
The upconversion fluorescent images on pig rib coming from the embedded HA:Yb/Ho powder in the drilled holes; (**a**) excited by 980 nm NIR laser pen, (**b**) excited by 980 nm NIR light under inverted fluorescence microscope. It demonstrates a possibility for long-term fluorescence tracking of implanted HA materials.

The HA:10Yb/1Ho particles were also used to fill in the drilled holes of fresh pig rib. The images in Fig. 4 show that the HA:Yb/Ho material filled in the holes can effectively emit its green fluorescence through the rib when irradiated by 980 nm NIR light, and the green fluorescence can be clearly observed by naked eyes (a) and under inverted fluorescence microscope (b). It provides a possibility for long-term fluorescence tracking of implanted HA biomaterials.

**Figure 5:**
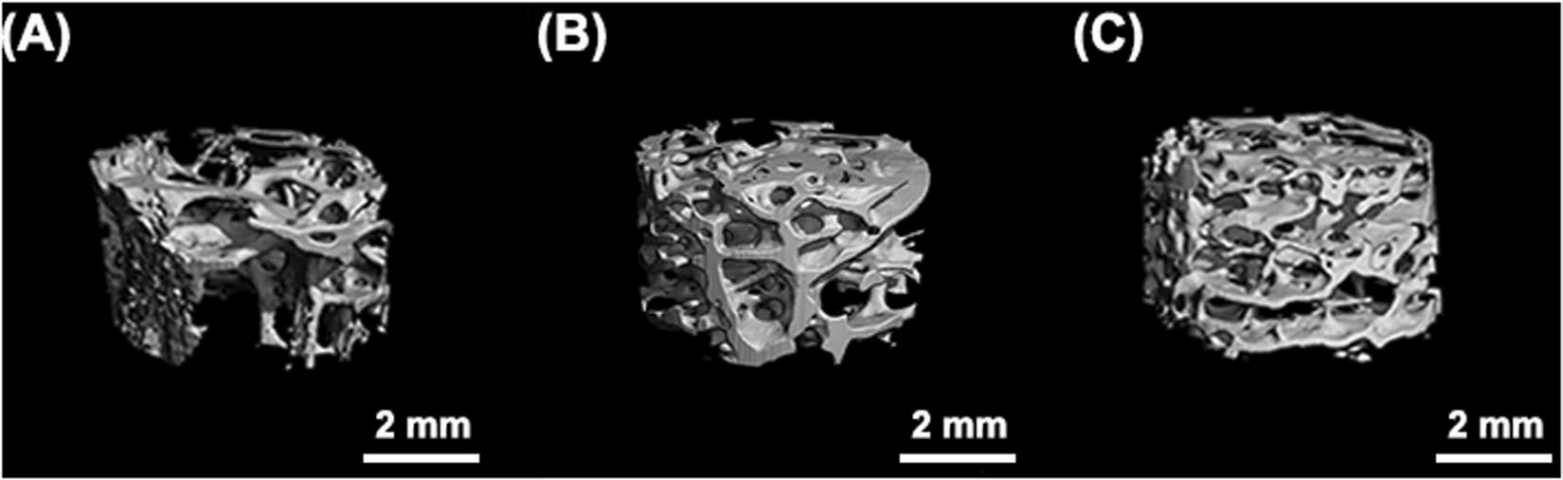
The 3D Micro-CT images of new bone tissue at 2 months (**A**), 4 months (**B**) and 6 months (**C**). The HA:Yb/Ho material has no negative effect on new bone formation and normal reconstruction, and can be directly used for bone repair.

**Figure 6:**
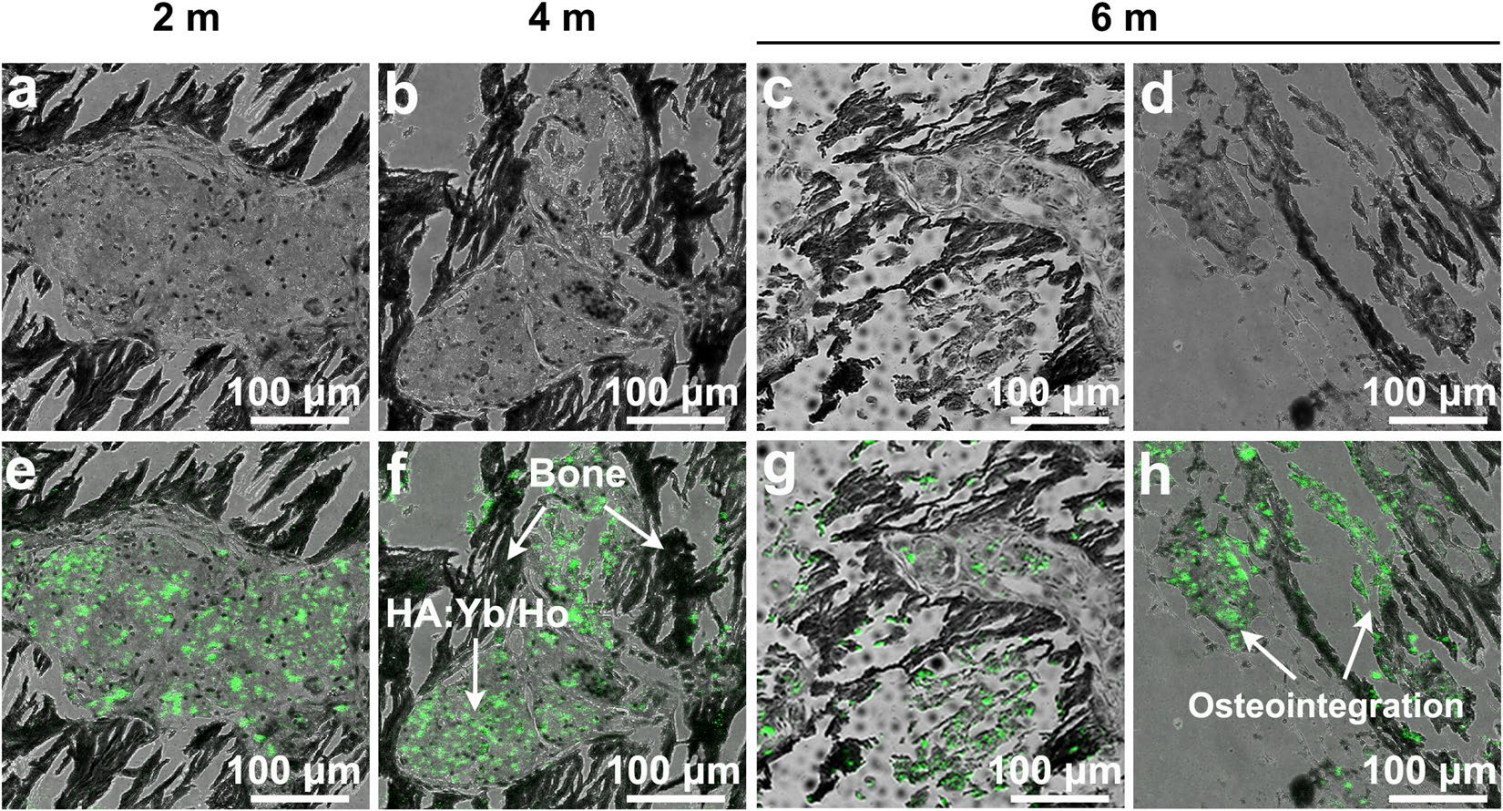
The confocal images of new bone tissue in black (**a**–**d**), the overlapping green upconversion fluorescent images of HA:Yb/Ho particles (**e**–**h**) under irradiation of 980 nm NIR light. The overlapping images show that the HA:Yb/Ho particles are mainly surrounded by the new bone tissue at 2 months (**e**), disperse into the region of new bone tissue at 4 months (**f**), mutual permeate (**g**) and form direct bonding contact (**h**) with bone tissue at 6 months.

The upconversion HA:10Yb/1Ho particles with an optimal emission intensity and anti-autofluorescence property were selected for implantation in the bone defects of rabbit femoral condyle, to investigate its effects on bone reconstruction and its superiority in long-term tracking. Figure 5 exhibits the 3D micro-CT images of new bone tissue at 2 months (a), 4 months (b) and 6 months (c). It can be seen that the volume of the regenerated bone tissue increases with the implantation time, which means the lanthanides doped HA:Yb/Ho material has no negative effect on new bone formation and normal reconstruction, and can be directly used for bone repair. Figure 6 shows the laser confocal images of new bone tissue, and the overlapping upconversion fluorescent images of the HA:Yb/Ho particles on the histological section of harvested samples at several time points. The newly formed woven bone tissues display a black color (Fig. 6(a–d)), while the HA:Yb/Ho particles present legible green fluorescence under irradiation of 980 nm NIR light in the overlapping images (Fig. 6(e–h)). The overlapping images clearly show the distribution of the implanted HA:Yb/Ho particles and the material-tissue interrelation. At 2 months, the HA:Yb/Ho particles are mainly surrounded by the new bone tissue (e). At 4 months, the particles have dispersed into the region of new bone tissue (f). At 6 months, new bone and particles not only permeate each other (g), but also form direct bonding contact or osteointegration (h). The results indicate that the Yb/Ho co-doped HA material has good *in vivo* biocompatibility, it is not only osteogenic but also suitable for long-term or lifelong *in vivo* tracking.

## Discussion

The entrance of the trivalent Yb^3+^ and Ho^3+^ ions into HA crystal structure by substituting the bivalent Ca^2+^ ions results in appearance of Ca^2+^ vacancies for the balance of charge, and slightly changes the binding energy of HA elements (Table 1). The difference in electronegativity between Ln^3+^ and Ca^2+^ may also influence the binding energy. However, the Yb^3+^ and Ho^3+^ doping will not change the hexagonal crystal structure of HA (Fig. 2c). For pure HA crystal (Ca_10_(PO_4_)_6_(OH)_2_), one unit cell contains 10 Ca^2+^ ions, six located at Ca(II) sites and four at Ca(I) sites^23,25^. The doping concentration of lanthanide ions shows distinct influence on the fluorescence emission of Yb^3+^/Ho^3+^ ions (Fig. 2a). For HA:Yb/Ho, small amount of Ho^3+^ doping (0.5 or 1) in all Yb^3+^/Ho^3+^ proportions seem better despite of the concentration of Yb^3+^ ions. The emission intensity of HA:10Yb/1Ho, HA:10Yb/0.5Ho and HA:30Yb/0.5Ho occupies the top three, and the 10Yb groups are more sensitive to Ho^3+^ dosage than the 30Yb groups. The highest intensity of 20Yb groups is for HA:20Yb/0.5Ho which is much lower than that of the top three. The optimal proportion for all HA:Yb/Ho groups in this experiment is Ca_10−x−y_/Yb_x_/Ho_y_ = 100/10/1 (i.e. HA:10Yb/1Ho), corresponding to a calculated molecular formula of (Ca_8.58_Yb_0.86_Ho_0.09_Vc_a0.47_)(PO_4_)_6_(OH)_2_ (Vc_a_ represents the Ca^2+^ vacancy). It can be seen from the formula that Ca^2+^ vacancies are present in the unit cell, i.e. there are 0.47 Ca^2+^ vacancies in per unit cell of HA:10Yb/1Ho. The presence of Ca^2+^ vacancies helps the charge balance of the doping HA lattice and ensures the successful substitution of the Ln^3+^ ions. In addition, the Ca^2+^ sites substituted by Ln^3+^ ions are mainly the Ca(II) sites reported by Kaur *et al*., due to the lower substitution energy and higher stability of the Ca(II) sites as compared to Ca(I) sites^26^. The unchanged hexagonal HA crystal structure enables the doping Yb^3+^ and Ho^3+^ ions to exhibit stable upconversion fluorescence emission.

The upconversion emission of HA:Yb/Ho material needs an external excitation of the NIR laser to convert lower energy photons into higher energy photons^27^. The green emission of the HA:Yb/Ho centered at 546 nm depends on the NIR energy transfer from Yb^3+^ ions to Ho^3+^ ions, and originates from the electron transitions of Ho^3+^ from (^5^F_4_, ^5^S_2_) level to ^5^I_8_ level_._ As shown in Figs S1 and 3, the NIR light and the HA:Yb/Ho material do have an advantage in avoiding tissue autofluorescence, and the NIR light shows deeper penetration into tissues than the UV light. Hence, the animal experiments were carried out by using the upconversion HA:10Yb/1Ho particles. The difference between the downconversion HA:Tb and the upconversion HA:Yb/Ho in fluorescence display is obvious after being covered by natural tissues. When the HA:Yb/Ho particles were irradiated by 980 nm NIR light through the covering tissues of fresh pig skin, muscle and bone, the excited upconversion fluorescence could pass through these upper tissues and displayed bright and undisturbed green luminescence. However, the green fluorescence of HA:Tb particles is invisible when irradiated by UV light through the same covering tissues. When the drilled holes of fresh pig rib were filled with the HA:Yb/Ho particles and irradiated by 980 nm NIR light (Fig. 4), the emitted green fluorescence of the upconversion particles could also pass through the rib, and be clearly observed by naked eyes or under inverted fluorescence microscope. The characteristic of the upconversion HA:Yb/Ho material provides a possibility and promising prospect for long-term *in vivo* investigation and applications.

The *in vivo* investigation shows that the lanthanides doped HA:Yb/Ho material owns an osteogenic capacity similar to pure HA material, and has no negative effect on bone formation and reconstruction. The regeneration of new bone tissue can normally progress with the implantation time as shown by the 3D micro-CT images in Fig. 5. In addition, the upconversion characteristic of the HA:Yb/Ho material shows obvious advantage not only on the fluorescence tracking, but also on revealing the material distribution *in vivo* and material-tissue interrelation, as shown in Fig. 6. The newly formed bone tissue can grow surrounding (6e) and penetrating the HA:Yb/Ho particles (6g), and form direct bonding contact with the particles (6h). Without the help of the upconversion fluorescence images from the particles (Fig. 6(e–h)), it is hard to distinctly identify the implanted particles from the images of Fig. 6(a–d). The good biocompatibility, osteogenic capacity and stable upconversion fluorescence of the HA:Yb/Ho material provide a feasibility to long-term or lifelong trace the implanted HA material or its composite scaffolds. Popular matrixes that can host rare-earth ions to trigger upconversion fluorescence also comprise NaYF_4_^28^, NaGdF_4_^29^, and LaPO_4_^30^, which have shown stronger fluorescence intensity, good cell imaging or *in vivo* targeting properties. However, the lanthanides-doped upconversion HA material should be more suitable for *in vivo* biomedical applications due to its similarity to the bone mineral and better affinity to human tissues.

Although the practical clinical applications of such osteogenic HA:Yb/Ho fluorescent material may depend on the progress of both material and instrumental technology^31,32^, the upconversion HA-based material does have specific advantage and will show its versatility in future biomedical applications. The research has demonstrated that the lanthanides doped upconversion HA material is suitable for long-term tracking of the material-tissue interrelation, the lanthanide ions coordinated in HA matrix will not affect the biocompatibility and osteogenesis of HA material, and the upconversion fluorescence of the HA:Yb/Ho material can effectively avoid the interference of tissue autofluorescence. For better use of the upconversion fluorescence in future *in vivo* tracking, it is highly desired to construct the upconversion material with its emission peaks in the “optical window”, from red region to NIR spectral range (600–1100 nm), to further reduce the strong light absorption and scattering of short-wave-length light below 600 nm by biological tissues^18^.

## Materials and Methods

### Materials and reagents

Lanthanide (Ln) compounds Yb(NO_3_)_3_ and Ho(NO_3_)_3_ in analytical grade were purchased from Shanghai Aladdin Co. Ltd., China. AR-grade Ca(NO_3_)_2_, Na_3_PO_4_, NaOH were obtained from Beijing Chemical Reagents Company, China. Other chemical reagents obtained from commercial routes were of AR grade and used without further purification.

### Synthesis of Ln-doped HA upconversion material

Dopants of Yb(NO_3_)_3_ and Ho(NO_3_)_3_ were selected to react with Ca(NO_3_)_2_ and Na_3_PO_4_ by a reaction formula (10−x−y)Ca(NO_3_)_2_ + xYb(NO_3_)_3_ + yHo(NO_3_)_3_ + Na_3_PO_4_ + NaOH → (Ca_10−x−y_Yb_x_Ho_y_Vc_a_)(PO_4_)_6_(OH)_2_. Where, Vc_a_ represents the Ca^2+^ vacancy. The molar ratio Ca_10−x−y_/Yb_x_/Ho_y_ was 100/(10~30)/(0.5~3), in which the ratios of Yb/Ho were set at 10/0.5, 10/1, 10/2, 10/3; 20/0.5, 20/1, 20/2, 20/3; and 30/0.5, 30/1, 30/2, 30/3, respectively. The synthetic route is as follows: Ca(NO_3_)_2_ (0.28 M, 28 mL) and Ln(NO_3_)_3_ (0.10–0.30 M Yb^3+^ +0.005–0.03 M Ho^3+^, 8 mL) aqueous solution was firstly placed in a Teflon-lined autoclave (100 mL) under magnetic stirring, then Na_3_PO_4_ (0.20 M, 28 mL) aqueous solution was added, the pH was adjusted to be 10 by NaOH. Afterwards, the mixture was agitated for 10 min, and hydrothermally treated at 160 °C for 10 h. After cooling to room temperature naturally, the white HA:Yb/Ho precipitates collected by centrifugation (5 min at 2700 × g) were fully washed by deionized water, and freezing-dried for 24 hrs. For comparison, wet synthesis of HA:Yb/Ho precipitate at 70 °C without hydrothermal treatment was also carried out. The dried HA:Yb/Ho powders were finally activated at 700 °C in air with a heating rate of 1 °C/min, to evoke their upconversion property.

### Characterization

The morphology of the HA:Yb/Ho particles was observed by transmission electron microscope (TEM) on an FEI Tecnai G2 T20 instrument at 200 KV. The X-ray diffraction (XRD) patterns were acquired with a PANalytical Empyrean equipment in the 2*θ* range from 20° to 60° with Cu Kα radiation (λ = 1.5406 Å). The Fourier transform infrared (FTIR) spectra were recorded in the transmission mode with a wavenumber range of 400–4000 cm^−1^ (Perkin-Elmer 6000). The binding energy data were measured by X-ray photoelectron spectroscopy (XPS) spectrum via AXIS Ultra DLD, Kratos, UK. The photoluminescence was recorded by a Hitachi F-7000 fluorescence spectrophotometer, attached with an external 0–2 W adjustable diode laser integrated with an optical fibre (Beijing Hi-Tech Optoelectronic Co., China). The concentration of Yb and Ho was measured by an inductively coupled plasma optical emission spectrometer (ICP-OES, Teledyne Leeman Labs).

### Cell proliferation assay

Osteoblastic MG63 cells together with the HA:Yb/Ho solution of 100 μg/mL were seeded onto 96-well tissue culture plates (Corning, USA) with a density of 2 × 10^4^ cells/well. Afterwards, the seeded samples were cultured in a humidified incubator (37 °C, 5% CO_2_), and the medium was changed every two days. MG63 cells cultured in F12 served as the control. After incubation for 1, 4, 7, and 11 days, the samples were evaluated with a CCK-8 assay and the optical density (OD value) of the solution was recorded using a microplate reader (PerkinElmer wallac 1420) at 490 nm to reflect the cell proliferation. The results were expressed as mean ± standard deviation (SD) from triplicate wells.

### Interference of tissue autofluorescence

Fresh tissues of pig skin, muscle and bone with a thickness of approximately 3 mm were used to investigate the interference of tissue autofluorescence. 0.05 g of the upconversion HA:Yb/Ho powder, and downconversion HA:Tb powder as a control, were covered by these fresh tissues (Fig. 1a) and observed via an inverted fluorescence microscope (Ti-U, Nikon, Japan) under irradiation of UV, blue, green or 980 nm NIR lights respectively. 0.02 g of the HA:Yb/Ho powder was also filled in the drilled holes (φ2 mm × 6 mm) of a fresh pig rib (Fig. 1b) to further investigate its upconversion fluorescence tracking effects under a 980 nm laser (Beijing Hi-Tech Optoelectronic Co., China), and the pump power was set at 0.5 W.

### *In vivo* experiments

All animals were used in accordance with protocol approved by the Research Ethics Committee of State Key Laboratory of Oral Diseases (Permit Number: WCHSIRB-D-2016-134) in compliance with all regulatory guidelines. Six adult New Zealand white rabbits were randomly divided into two groups for implantation (n = 3 in each group). After shaving and disinfection of the hind limbs, a cylindrical bone defect (φ6 mm × 5 mm) was drilled on the distal femoral condyle (Fig. 1c). The HA:Yb/Ho powder was implanted into the defects and harvested with surrounding tissue at 2, 4 and 6 months after implantation. The harvested samples were fixed in 10% formalin, dehydrated through gradient ethanol, then embedded with PMMA and cut into thin sections for image observation via laser confocal microscopes (Nikon A1R MP+, Japan) under 980 nm. Micro-CT (VivaCT 80, SCANCO Medical AG, Switzerland) was also employed to determine whether the lanthanides doped HA:Yb/Ho material would affect the bone reconstruction.

All experimental procedures were conducted in conformity with institutional guidelines for the care and use of laboratory animals. All mice were sacrificed with an intraperitoneal injection of a lethal dose of 10% chloral hydrate, and all efforts were made to minimize suffering.

### Statistical analysis

All results are expressed as mean ± standard deviation (SD). Statistical comparisons between groups were analyzed by using one-way ANOVA test. A value of P < 0.05 was considered to be statistically significant.

## Conclusion

The HA:Yb/Ho material with a formula (Ca_8.58_Yb_0.86_Ho_0.09_Vc_a0.47_)(PO_4_)_6_(OH)_2_ could exhibit optimal upconversion emission under 980 nm NIR excitation. The upconversion material can effectively avoid the autofluorescence of various natural tissues, like the skin, muscle and bone. It owns good biocompatibility and an osteogenic capacity similar to pure HA material, and has no negative effect on bone formation and reconstruction. The lanthanides-doped upconversion HA:Yb/Ho material will exhibit its versatility in future biomedical applications for repair of bone and teeth, and *in vivo* lifelong tracking.

## Electronic supplementary material

Supplementary Information
