# Supplementary material for: Investigation on Anti-Autofluorescence, Osteogenesis and Long-Term Tracking of HA-Based Upconversion Material
Source: Sci Rep. 2018 Jul 26;8:11267. doi: 10.1038/s41598-018-29539-8 (PMC6062553; doi:10.1038/s41598-018-29539-8)
Supplement: Supplementary file 1 — Supplementary Information [file 41598_2018_29539_MOESM1_ESM.doc]

Supplementary Information

Investigation on Anti-Autofluorescence, Osteogenesis and Long-Term Tracking of HA-Based Upconversion Material

Xiyu Li^1^, Qin Zou^2^, Wei Li^1*^ and Haifeng Chen^3*^


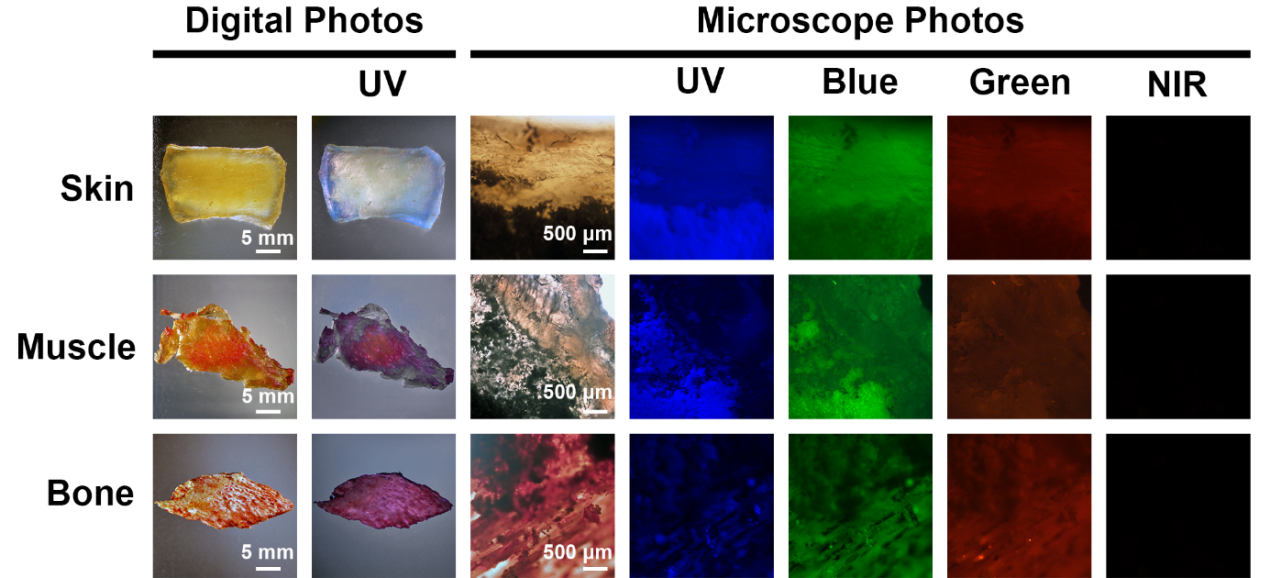
Figure S1. The digital photos and the microscopic photos of natural tissues of approximately 3 mm in thickness with underlying downconversion HA:Tb powder under irradiation of various lights. It shows that natural tissues (skin, muscle and bone) display the blue, green or red autofluorescence under irradiation of UV, blue or green lights respectively. The green fluorescence of the underlying HA:Tb powder cannot be observed. However, there is no tissue autofluorescence under irradiation of 980 nm NIR light.
